# Stem Cell-Based Therapeutic Strategies in Diabetic Wound Healing

**DOI:** 10.3390/biomedicines10092085

**Published:** 2022-08-25

**Authors:** Meng-Chien Willie Hsieh, Wei-Ting Wang, Chuang-Yu Lin, Yur-Ren Kuo, Su-Shin Lee, Ming-Feng Hou, Yi-Chia Wu

**Affiliations:** 1 Department of Surgery, Division of Plastic Surgery, Kaohsiung Medical University Hospital, Kaohsiung 80708, Taiwan; 2 Department of Plastic Surgery, Kaohsiung Municipal Ta-Tung Hospital, Kaohsiung 80145, Taiwan; 3 Department of Biomedical Science and Environmental Biology, College of Life Science, Kaohsiung Medical University, Kaohsiung 80708, Taiwan; 4 Department of Surgery, School of Medicine, Kaohsiung Medical University, Kaohsiung 80708, Taiwan; 5 Regenerative Medicine and Cell Therapy Research Center, Kaohsiung Medical University, Kaohsiung 80708, Taiwan; 6 Department of Surgery, Kaohsiung Municipal Siaogang Hospital, Kaohsiung 81267, Taiwan; 7 Department of Surgery, Division of Breast Oncology and Surgery, Kaohsiung Medical University Hospital, Kaohsiung 80708, Taiwan

**Keywords:** diabetic wound healing, diabetic foot ulcers (DFU), embryonic stem cells (ESCs), induced pluripotent stem cells (iPSCs), mesenchymal stem cells (MSCs), adipose-derived stem cells (ASCs)

## Abstract

Impaired wound healing and especially the “all-too-common” occurrence of associated diabetic foot ulcers (DFU) are becoming an increasingly urgent and deteriorating healthcare issue, which drastically impact the quality of life and further heighten the risks of infection and amputation in patients with diabetes mellitus. Amongst the multifactorial wound healing determinants, glycemic dysregulation has been identified to be the primary casual factor of poor wound healing. Unfortunately, current therapeutic modalities merely serve as moderate symptomatic relieves but often fail to completely restore the wound site to its pre-injury state and prevent further recurrence. Stem cell-based therapeutics have been employed for its promising potential to address the root of the problem as they not only exhibit the capacity for self-renewal and differentiation towards multiple lineages, but also have been disclosed to participate in mediating variant growth factors and cytokines. Herein we review the current literatures on the therapeutic benefits of using various kinds of stem cells, including embryonic stem cells (ESCs), induced pluripotent stem cells (iPSCs), mesenchymal stem cells (MSCs), and adipose-derived stem cells (ASCs) in diabetic wound healing by searching on the PubMed^®^ Database for publications. This study shall serve as an overview of the current body of research with particular focus on autologous ASCs and the laboratory expandable iPSCs in hope of shedding more light on this attractive therapy so as to elevate the efficacy of wound healing that is almost always compromised in diabetic patients.

## 1. Background

A vast array of therapeutic modalities exists solely for the field of wound healing. For chronic and difficult wounds, such as diabetic wounds, regenerative medicine is rapidly emerging largely due to its immense potential on enhancing and restoring tissue repair. Regenerative medicine in wound healing hinges on the regulation and modulations of growth factors, stem cells, biomaterials and tissue engineering, or an overlapping combination of the above [1]. Despite the treatment guidelines and even the adjunctive therapies that are available, current strategies are still insufficient to overcome the burden of this disease [2]. More specifically, diabetic wound repair is often severely delayed by the underlying ischemia, infection, neuropathy, and metabolic disorders [3]. Advancement in the knowledge of each phase of the wound healing process has aided the development of regenerative medicine, particularly stem cell therapy, to reduce the morbidities associated with diabetic foot ulcers (DFU). This study serves to review the existing literature regarding diabetic foot ulcers, the wound healing process, the regulatory growth factors and cytokines, and the stem cell therapies currently available or in study, so as to shed light on the potential therapeutic targets for a more precise treatment and the future development of stem cell-based regenerative medicine.

### 1.1. Diabetes Mellitus

As the world evolves to be increasingly developed with the constant advancement of technology throughout human history, our diet structure has correspondingly become highly refined and heavily laden with processed food, which is a main culprit for chronic diseases with deadly consequences such as diabetes mellitus. Epidemiological studies estimated the global diabetes prevalence to be 9.3% (463 million people) in 2019, which is projected to rise to 10.2% (578 million) by 2030 and 10.9% (700 million) by 2045 [4,5]. Moreover, the World Health Organization estimated diabetes to be the 7th of the top 10 causes of death in 2030 [6]. This growing incidence of diabetes is reflected on the increasing burden on the patients, the family, the healthcare system, and therefore, the economy. In the US alone, the expenditure associated with DM was $245 billion USD in 2012, which was a drastic 41% increase compared with the amount spent in 2007 [7].

### 1.2. Diabetic Wounds and Diabetic Foot Ulcers (DFU)

The wound is defined as a disruption of the integrity of the skin, mucous membrane or tissue [8]. Wounds can further be classified as acute, which heal within three weeks, or chronic, the healing of which extends over six weeks, depending on the duration of occurrence [9]. In contrast with acute wounds, chronic wounds exhibit a hindered healing process (longer than 12 weeks) due to persistent pathological inflammation [10]. In general, chronic wounds are categorized as diabetic, pressure or vascular ulcers, which are often the outcome of a disease complication [1].

One of the major challenges of patients suffering from diabetes is the increased risk of developing chronic wounds that fail to heal [11]. The annual expenditure on chronic wound treatment is even estimated at $31.7 billion [12]. Amongst the chronic wounds associated with diabetes, DFU, defined as a full-thickness wound below the ankle, is one of the most common and hard-to-treat complications [13]. The alarming rise in DFU has been reported to affect as many as more than a quarter of diabetic patients [14].

#### Pathogenesis of Diabetic Wound and DFU

The underlying causes of diabetes-related non-healing wounds could be multifactorial, such as deficiency of growth factors, dysregulated cell function, microvasculopathy and glycemic dysregulation. More specifically, the tremendous difficulty in treating the wounds of diabetic patients can be attributed to their inability to regulate glucose metabolism, which leads to a hyperglycemic condition that further impedes every phase of wound healing [6]. Of the various cells involved in wound healing, such as neutrophils, monocytes, macrophages, keratinocytes, T- and B-cells, mast cells and endothelial cells, macrophages, which are a major contributor to wound healing, undergo dysregulated polarization and modulation as a result of hyperglycemia and the associated oxidative stress [15,16]. In addition, impairment in neutrophil function, keratinocyte and fibroblast migration and proliferation, and persistent production in pro-inflammatory cytokines all lead to the non-healing of diabetic wounds [17,18]. Hyperglycemia also results in the glycation of hemoglobin and structural changes of red blood cell membrane, which further leads to a hypoxic condition in wound site and delayed wound healing [19].

Apart from the hypoxic state, the ischemia characteristics of diabetic wounds are largely due to vascular compromise and an ongoing inflammation that prevents the formation of mature granulation tissue and optimal wound tensile strength [17,20]. The narrowing or blockage of distal limb arteries is a common feature found in diabetic patients. The endogenous compensating mechanisms, including capillary growth (angiogenesis) and the development of collateral arterial vessels (arteriogenesis), usually occur with the occlusion of arterial vessels in diabetic non-healing wounds. According to the guidelines reported by International Working Group on the Diabetic Foot (IWDGF) in 2011, up to 50% of diabetic patients have been diagnosed with peripheral arterial disease (PAD). Therefore, the mobilization of hematopoietic stem cells and the pro-angiogenic cells from bone marrow to the peripheral circulation is inhibited in diabetic patients [21], which further prolonged poor glycemic control-caused non-healing in diabetes mellitus.

Furthermore, physiologic dysfunction such as increased ratio of matrix metalloproteinase-9 (MMP-9) in serum, reduced collagen accumulation and dysregulation in the ratio of collagen types, decreased neuropeptide expression, insufficient production of thrombin-activatable fibrinolysis inhibitor, glycation of PDGF, as well as extracellular matrix (ECM) degradation as regulated by MMP, all contribute to delayed healing in diabetic patients [6,22].

## 2. The Process of Wound Healing

During the process of wound healing, there are three distinct but overlapping phases, namely inflammation, proliferation and remodeling, are highly coordinated and intricately intertwined. The biological process of dermal wound repair is an exceedingly complex yet normal activity involving interaction between cells and inflammatory mediators [23]. Depending on the injury mechanism, wound healing is a continuous progression involving a coordinated interplay amongst epidermal and dermal cells, the extracellular matrix, orderly angiogenesis and plasma-derived proteins, which are regulated by cytokines and growth factors [24,25]. Any disruption and dysregulation play crucial roles in wound healing.

### 2.1. Inflammation

As the very first stage in wound healing, inflammation, which consists of vascular response (hemostasis) and cellular response (inflammation), takes place immediately after a wound is created so as to prevent and lower the risk infection [26]. In the inflammatory response, fibrin clot and damaged tissue-released cytokines such as transforming growth factor (TGF-β), platelet-derived growth factor (PDGF), epidermal growth factor (EGF), fibroblast growth factor (FGF), and interleukin 8 (IL8) play crucial roles in this stage [27]. These inflammatory cytokines serve as chemotactic signals to recruit neutrophils to the wound to ingest and destroy foreign particles such as bacteria and apoptotic cells of damaged tissue via their phagocytotic activity before being replaced with macrophages approximately three days after injury [28]. 

### 2.2. Proliferation

The proliferative phase of wound healing is to decrease the lesion size via contraction and fibroplasia [29]. Between 2 to 10 days after the presence of a wound, proliferation kicks in to call forth keratinocytes to the compromised dermis, closely followed by angiogenesis. Throughout this phase, granulation tissue starts to fill the wound via capillary sprouts that are accompanied by fibroblasts and macrophages. Several focal factors also participate during this stage, including fibroblast growth factor and vascular endothelial growth factor [30]. Towards the end of this phase, macrophages, derived from circulating monocytes originating in the bone marrow, transform into myofibroblasts, which are contractile cells that crucially contribute to the contraction of wounds [31].

### 2.3. Remodeling

Remodeling is the third phase of wound repair and is perceived as the most important clinically [32,33]. Through the rearrangement, degradation and reformation of ECM, the aim of this phase is to reach scar maturation and maximum tensile strength [29]. Granulation tissue becomes remodeled and collagen fibers increase in concentration in this final phase of tissue structure recovery [34]. This process is regulated by various growth factors such as TGF-β1 and FGF, in addition to a decreased synthesis of chemokines by cytokines such as IL-10 [29].

### 2.4. Regulatory Growth Factors and Cytokines in Wound Healing

During the process of wound healing, multiple growth factors and cytokines have been documented closely interrelated in almost all stages not only in the normal healing process, but also playing an indispensable role in impaired wound healing. These essential regulators mediate inflammatory responses, induce proliferation and migration of various cell types, stimulate angiogenesis, and maintain the balance of the extracellular matrix remodeling, which are all essential and required in wound healing [35]. Herein, we highlight the key growth factors and cytokines that have been reported to play crucial roles in diabetic wound healing (Table 1).

#### 2.4.1. Tumor Necrosis Factor-Alpha (TNF-α)

Overexpression of pro-inflammatory mediators and continuous inflammation are some of the causes underlying delayed wound healing in patients with diabetes. Many previous investigations have disclosed the role of increased tumor necrosis factor alpha (TNF-α), a member of TNF superfamily, in diabetic impaired wound healing [36,37]. High glucose levels enhance M1 macrophages polarization and prolonged the infiltration of this pro-inflammatory macrophage to abnormally release excess TNF-α, which further induces several downstream inflammatory signals and extended inflammation phase, leading to delayed wound healing in diabetes. Increased TNF-α was also found to induce the expression of tissue inhibitor metalloproteinases-1 (TIMP-1) which impaired keratinocytes migration for wound re-epithelialization. Using a TNF-α antagonist was evidenced to improve wound healing in STZ-induced diabetic rats [36]. Furthermore, a prolonged M1 macrophages polarization means a shortened M2 state of macrophages. M2 macrophages not only secrete anti-inflammatory cytokines, but also promote wound healing by participating in wound closuring, angiogenesis, re-epithelialization, and tissue regeneration [37].

#### 2.4.2. Transforming Growth Factor Beta (TGF-β)

In mammals, macrophage-, keratinocyte-, fibroblast-, and platelet-produced TGF-β1 play crucial roles in wound healing among 3 TGF-β isoforms (TGF-β1-3) [38]. In general, TGF-β binds to two transmembrane dual specificity TGF-β receptors (TβRI and TβRII) to phosphorylate downstream regulatory cytoplasmic proteins with its strong serine/threonine kinase activity [39]. TGF-β1 not only attracts chemokines and mediates various immune cells, but also serves as an antagonist to suppress the ability of extent immune response in injured tissues [40]. 

Furthermore, TGF-β was revealed to participate in re-epithelialization, angiogenesis, and ECM synthesis in the proliferative phase of wound healing. TGF-β promotes proliferation and migration of epithelial cells and play crucial roles in inducing angiogenesis in the proliferative phase of the wound healing by promoting vascular endothelial growth factor (VEGF) expression [41]. TGF-β also recruits fibroblasts from adjacent tissues including the dermis, blood vessels and bone marrow for ECM synthesis [42,43,44]. TGF-β1 regulates αSMA expression in fibroblasts, which leads the cells acquire a contractile phenotype for remodeling, the final phase of wound healing after the injury [43,45]. In diabetic animal models, a single intravenous injection of TGF-β1 was demonstrated to be sufficient for improving wound healing with increased endothelial cell proliferation and an expressive amount of well-organized collagen fibrils [46]. 

#### 2.4.3. Epidermal Growth Factor (EGF)

Epidermal growth factor (EGF), the most investigated growth factor in wound healing, was firstly isolated and purified from salivary glands in 1962. EGF stimulates cell proliferation and differentiation to regulate various downstream signaling cascades by binding to its cognate receptor (EGFR) which is a kind of receptor tyrosine kinases. The reduction of EGF has been documented to be associated with diabetes mellitus and was considered as one of the reasons of diabetic impaired wound healing [47]. Therefore, administration of EGF has been attempted for treating poor healing diabetic wounds. For instance, combining polyurethane with EGF-conjugated silk fibroin had been revealed to exhibit an anti-inflammatory response which further improves wound healing in diabetic rats [48]. Clinically, the incorporation of EGF with nano silver dressing was also evidenced to significantly shorten the time for wound repair with elevated granulation tissue formation in patients suffering from DFU [49].

#### 2.4.4. Vascular Endothelial Growth Factor (VEGF)

The therapeutic effects of VEGF in wound healing have been well recognized owing to its vasculogeneic and angiogeneic ability. It has been widely investigated that VEGF serves as a signaling growth factor to stimulate angiogenesis by interacting with multiple tyrosine kinase receptors (the VEGFRs) including VEGF receptor-1 (VEGFR-1) and VEGF receptor-2 (VEGFR-2). When VEGF binds to the receptor, VEGFR dimerizes and result in kinase activation to trigger variant downstream signaling cascades [50] to inhibit apoptosis and promote cell proliferation and migration of fibroblasts and endothelial cells. Activated VEGFR further promotes neovascularization, re-epithelialization, and collagen deposition [51]. Even in diabetes-related chronic wounds, using an artificial three-dimensional VEGF-bound collagen scaffold was proven to improve vascularization and enhance wound healing in diabetic animals with increased VEGF found in the granulation tissue [52].

#### 2.4.5. Platelet-Derived Growth Factor (PDGF)

Platelet-derived growth factor (PDGF), a platelet-secreted biochemical mediator, has been confirmed to play critical roles throughout all stages of wound healing and further promotes wound healing [53]. PDGF stimulates its two tyrosine kinase receptors, including the PDGFRα and the PDGFRβ to trigger several signaling pathways to regulate multiple cellular and developmental responses [54]. In terms of initiation and propagation in tissue repair, PDGF not only exhibits chemotactic and stimulatory effects, but also triggers mitogenesis, angiogenesis, which are able to promote cell proliferation. 

In case of PDGFRβ targeted deletion in dermal fibroblasts, an approximately 85% reduction of granulation tissue mass was observed during the process of wound healing [55]. By contrast, the administration of exogenous PDGF into a collagen-chitosan composite to mimic the ECM in wounds was demonstrated to enhance the expression of antioxidants and lipid peroxide in the treated tissues, which exhibited significant re-epithelialized tissues at the wound site and an accelerated wound healing process [56]. Notably, the administration of PDGF was reported to be able to also regulate oxidative balance in the process of diabetic wound healing in vivo. A reduction of NOx levels in the early stage and an upregulated NOx levels in the late stage of wound healing was evidenced [57].

#### 2.4.6. Fibroblast Growth Factor (FGF)

FGF is a type of polypeptide growth factor with cell signaling capacity, which widely exists in almost all organs and tissues. FGF functions as a controller of cell proliferation, differentiation and survival, angiogenesis, and keratinocyte organization, which are all highly critical to the wound healing processes [58]. 

Importantly, FGFs are angiogenic factors more potent than PDGF and VEGF [59]. FGFs not only stimulate angiogenesis, but also induce fibroblast proliferation to form granulation tissues. The newly formed granulation tissues then fill the dead space and the wound cavity in the initial stages of wound repair [35]. Several studies revealed that some FGF subtypes such as the aFGF, bFGF and FGF 15/19 subfamilies, may benefit the healing process of diabetic wounds. For instance, administration of aFGF was found to significantly multiply the number of capillaries in diabetic wounds, thus improving the healing of diabetic ulcer in diabetic rats [60]. FGF-19 had been observed to be upregulated in the serum of diabetic patients and believed to endocrinologically regulate major metabolic processes of blood glucose, suggesting it to be a potential therapeutic target of targeted therapies for the treatment of diabetic wound healing [61].

#### 2.4.7. Interleukins (IL)

Interleukins (IL) are cytokines primarily secreted by white blood cells (leukocytes). There are over 50 interleukins and specific related proteins have been identified in which most of them serve as important inflammatory molecules responsible for the immune system [62], thus playing crucial roles in wound healing. Interestingly, while some elevated IL levels such as IL-1β have been determined in the development of insulin resistance and diabetic non-healing accompanying with prolonged inflammation [63], there are still some ILs that exhibit beneficial effects on promoting diabetic wound repair. IL-20, IL-22, and IL-24 were all documented to accelerate diabetic wound healing [59]. 

Among these, treatment with IL-22 for DFU has been shown to enhance the level of VEGF and inhibit diabetic keratinocyte differentiation with a significantly shortened process of wound healing [64]. Mechanistically, IL-22 was identified to encourage vascularization, re-epithelialization, and granulation, which further promote wound healing of diabetic foot ulcers with a more effective therapeutic influence than PDGF and VEGF [59]. Furthermore, IL-25 has also been evidenced to be involved in tissue regeneration and glucose homeostasis regulation, which are crucial in diabetic wound healing. 

Topical treatment with exogenous IL-25 recombinant protein was confirmed to improve angiogenesis and vascular remodeling in the wound site and thereby ameliorate prolonged diabetic non-healing in STZ-induced diabetic mice [65]. Altogether, these studies all highlight the important regulatory roles of ILs in the process of wound repair even in diabetic wounds with poor healing.

## 3. Current Diagnostic Guidelines and Treatment Strategies of Diabetic Wound Healing

As nearly a quarter of diabetic patients are at risk for developing diabetic foot ulcers, it is of the utmost importance to establish the etiology at the outset so as to select the proper therapy to provide thereon [66]. The foremost clinical survey is to determine the presence of any clinically significant arterial diseases by arranging the ankle-brachial index (ABI), which is a quick and noninvasive test to check for PAD [67,68]. An ABI greater than 0.9 can, in general, rule out PAD, whereas an ABI < 0.9 suggests the presence of PAD [69]. Moreover, anatomic and physiologic data can be collected by measuring the transcutaneous oxygen pressure, hyperspectral imaging analysis, color duplex ultrasound scanning [70,71,72].

Once the etiology has been established, the central tenet for treating DFU is to achieve tissue healing without compromising functionality and weight bearing for ambulation [73]. Common strategies for managing diabetic wounds, especially DFUs, consist of debridement, infection control, lower limb revascularization, and pressure off-loading [74]. When profound ischemia is evident, infection control takes priority over limb revascularization. While inpatient care is suggested for a patient’s extensive tissue injury, pedal pulses and uncontrolled infection, intervention with vascular surgery or emergent debridement are necessary. Other adjunctive therapies have also been proven to accelerate wound healing in DFU, including some topical antimicrobial agents such as silver sulfadiazine and murpicin, growth factors, biologic medications, negative-pressure therapeutics, and administration of hyperbaric oxygen [75]. Once both medical and surgical treatment have been provided, a multidisciplinary team consisting of the primary physician, diabetologist, nurse educator, prosthetist, and home care health worker is crucial to providing total patient care and addressing the multifactorial problems in cases of invasive infection, diabetic peripheral neuropathy, or impaired tissue perfusion with and without gangrene [73].

## 4. Stem Cell Therapy in Diabetic Wound Healing

On the grounds of the currently available treatment modalities that are still limited in therapeutic efficacy, growing attention and focus have gravitated towards the application of stem cell therapy for chronic wound healing. In definition, stem cells are unspecialized cells of the human body that are distinguished by their potent capability for self-renewal, replication, and differentiation into other cell types [76]. The various cytokines and growth factors secreted by stem cells have been demonstrated to play a central role in immunomodulatory activities and regeneration, which are often biologically deficient in chronic wounds, thereby making stem cells particularly promising and fit as a treatment option [77,78]. Herein, we discuss both the benefits and limitations of the major stem cell types applied in diabetic wound healing (Table 2).

### 4.1. Embryonic Stem Cells (ESCs)

Embryonic stem cells (ESCs) are pluripotent stem cells which were first established from the inner cell mass of mouse embryo in 1981 and are able to differentiate into various cell types including neural cells, blood cells, adipocytes, chondrocytes, muscle cells, and skin cells [79]. This remarkable regenerative potential that has garnered increased attention to be employed in cutaneous repair and wound healing. An ex vivo induction of human ESCs into basal keratinocytes was evidenced to be fully functional and enabled the construction of a pluristratified epidermis to facilitate wound healing in immunodeficient nude mice [80]. Furthermore, ESCs were also demonstrated to be safe and clinically beneficial (at least symptomatic improvements) in variant diseases including spinal cord injury, type 2 diabetes mellitus, emphysema, multiple sclerosis, and Lyme disease, without any adverse events being reported [81,82,83,84]. 

In diabetic wound healing, direct transplantation of ESCs by topical injection into skin wounds exhibited a significant improvement of wound healing with remarkably higher EGF and VEGF levels in diabetes-induced rats [85]. Furthermore, extracts of acellular embryonic stem cells were proven to facilitate wound closure, contraction and re-epithelialization, and angiogenesis with immunoregulatory and anti-inflammatory properties in diabetic mice [86]. The application of ESC-derived keratinocytes has also been proven to accelerate diabetic wound healing in STZ-induced mice in vivo with immunohistochemically increased activities of cytokeratin 8 and cytokeratin 14 [87]. In general, ESCs could be the most favorable type of stem cells for tissue regeneration, especially for diabetic wound healing, due to its self-renewal ability and the almost unlimited potential in differentiating into all classes of adult lineages. However, due to ethical, religious, and legal limitations, the application of ESCs still remains controversial.

### 4.2. Induced Pluripotent Stem Cells (iPSCs)

Induced pluripotent stem cells (iPSCs) are the most modern class of pluripotent stem cells, which was just established by Takahashi et al. in 2006 [88]. The introduction of four genes (Oct-3/4, Sox2, c-Myc, and KLF4) into murine somatic cells derived from mouse tail had been observed to be capable of reprogramming the already differentiated mature cells back to an embryonic state. In the successive year, iPSCs were produced using human cells, which exhibited a high similarity to ESCs in morphology, proliferation ability, and pluripotency [89], and were able to theoretically differentiate into all types of cells. This revolutionary technology makes utilizing “autologous” pluripotent stem cell populations in regenerative medicine possible without the major restrictions of employing ESCs due to ethical concerns and the possibility of immunological rejection. In regard to tissue regeneration and wound healing, several preclinical studies have reported that iPSCs-induced mesenchymal stem cells (iMSCs) contribute to the regeneration and improvement of blood vessel, periodontal tissue, liver, heart, chondrocyte, skeletal muscle, and cutaneous wound [90]. 

Recently, Yang et al. also induced human iPSCs to differentiate into MSCs (iMSCs) and demonstrated that iMSCs were able to improve mucosal healing in mice with colitis, accompanied with enhanced epithelial cell proliferation [91]. In addition to the proliferation of epithelial cells, keratinocytes, fibroblast and the like, blood supply is vital to the process of wound healing. Even the iMSCs-derived conditioned medium was revealed to effectively accelerate wound closure and enhance angiogenesis in vivo [92]. Furthermore, the conditioned medium of human iPSCs-derived smooth muscle cells (hiPSCs-SMCs) was also determined to present with increased angiogenic & regenerative cytokines [93]. These hiPSCs-SMCs-embedded 3D collagen scaffolds were proven to promote angiogenesis and accelerate diabetic wound healing in diabetic nude mice [93]. However, despite the promising benefits of iPSCs in regenerative therapeutics and wound healing, cancer risk development due to the use of retroviral vectors, low cell reprogramming efficiency, and high processing costs still pose as major challenges in iPSC-based regenerative therapy and diabetic wound healing.

### 4.3. Mesenchymal Stem Cells (MSCs)

MSCs are the most researched due to their safety and ease of harvest from dermal and adipose tissue [1]. As a prototypic adult stem cell with the capability to self-renew and differentiate, mesenchymal stem cells (MSCs) have been reported for their numerous benefits in treating several disorders as they participate in organ homeostasis, successful aging, and especially wound healing [94,95]. This multipotent stem cell population can be harvested from several different tissue sources, such as the bone marrow, adipose tissue, amniotic fluid, and dermis [33]. MSCs are characterized by their outstanding capacity for wound healing as a result of their propensity for tissue regeneration and immunomodulation via paracrine signaling and their exceptional mobility to travel sites of injury [96,97,98]. In consequence, accelerated wound closure has been observed due to stimulated neovascularization and re-epithelization through an enhanced migration of dermal fibroblast and keratinocytes [99,100]. 

In diabetes-related non-healing, MSCs have been evidenced to accelerate diabetic wound closure by modulating the inflammatory environment, encouraging cell proliferation for re-epithelialization, and promoting angiogenesis and granulation tissue formation [101]. Transplantation of MSCs accelerated wound closure, ameliorated clinical parameters, and avoided amputation complicated by diabetic foot ulcers [102]. Additionally, EGF-stimulated MSCs was confirmed to restore the impaired blood flow by stimulating the neovascularization through the modulation of VEGF/VEGF receptor pathways in diabetic mice [103]. 

Importantly, the clinical value and strength of the application of MSCs is particularly evident as it does not trigger any immune response by inhibiting the proliferation of B- and T-cells and maturation of monocytes while stimulating the production of regulatory T-cells and M2 macrophages [104,105]. However, despite the promising potential of employing MSCs in diabetic wound healing, there are still some concerns of using this type of stem cells. Decreased self-renewal ability and limited differentiated capacity are the major limitations of MSCs. Compared to pluripotent ESCs and iPSCs, multipotent MSCs can only differentiate into tissue-forming cell lineages [106]. If the bone marrow is selected as the source of MSCs, the invasive nature of the isolation process can pose as a daunting challenge that further deters the patients in need.

#### Adipose-Derived Stem Cells (ASCs)

Amongst MSCs, highly abundant and easily obtainable adipose-derived stem cells (ASCs), have been well recognized to be another promising candidate applying in diabetic wound healing [107]. ASCs share a high similarity of phenotypic and functional characteristics with bone marrow-derived MSCs as ASCs not only present with the capacity to differentiate into diverse cell lineages, such as adipogenesis, chondrogenesis, osteogenesis, and myogenesis [108], but also promote cell growth and accelerate wound healing by secreting a multitude of growth factors including VEGF, FGF, and HGF [109]. The administration of ASCs has been evidenced in accelerating diabetic wound healing in various animal models. We have discovered that local administration of ASCs into the wound margin triggered angiogenesis and increased tissue regeneration, which further promoted diabetic wound healing through paracrine and autocrine mechanisms including an increase in von Willebrand factor and VEGF in streptozotocin (STZ)-induced diabetic rodent model [110]. We have also reported that the exogenous replenishment of the cutaneous CTACK/CCL27 at least partially restored the migration ability of ASCs, which was diminished in a high-glucose condition, and might eventually improve diabetic wound healing [107]. These studies strongly suggest that delayed wound healing in patients suffering from diabetes may be attributed to suppressed expressions of growth factors and regulatory cytokines such as CTACK/CCL27, which further lead to a deficient stem cell migration to the wound sites, thus implicating that the exogenous administration of ASCs contribute to diabetic wound healing. Similar to MSCs, lower self-renewal and differentiating abilities with a short lifespan are also defects inherent to ASCs. Moreover, donor specificities including age, gender, and healthy conditions, all influence the potency and quality of ASCs [106]. In sum of the aforementioned strengths and weaknesses, ASCs are still a highly and more favorable candidate for stem cell therapeutics.

## 5. Future Directions

To overcome the existing limitations of employing stem cells in clinical cell therapeutics, a cell-free system would be an attractive strategy. Several animal and human studies have reported that bone marrow-released stem cells are deficient or impaired in diabetic patients [111]. This may explain for the poor wound healing frequently observed in diabetic patients as deficiency of bone marrow-derived progenitor cells induce defect in classical processes including cell proliferation, migration, neovascularization, and mesenchymal formation in wound healing. Both MSCs and ASCs exhibit great immunomodulation abilities via paracrine signaling without ethical problems and immunological rejection. Studies have shown that MSCs improved diabetic wound healing via their paracrine signaling capability. Exosomes derived from MSCs induced cell proliferation, migration and angiogenesis of diabetic wound fibroblasts in vitro [112]. 

Importantly, merely the cell-free exosomes secreted by ASCs (ASCs-Exos) have been revealed to be sufficient to accelerate diabetic wound healing. ASCs-Exos not only promoted proliferation and angiogenesis in endothelial progenitor cells in a high-glucose environment, but also significantly reduced ulcerated regions in the feet of diabetic rats [113]. This cell-free model further highlights an ASC-based strategy in diabetic wound healing to be safer and highly promising.

## 6. Conclusions

The multifactorial causes of diabetic wounds and the impaired wound healing ability in diabetic patients remain a formidable challenge and financial burden to the health system and for all medical practitioners around the world. Due to the aging worldwide population and the increasing incidence of chronic wounds that ensue, medical progress in the field of wound therapeutics is in dire need. Given the fact that stem cells exhibit immense potential in regenerative medicine and possess the regulatory abilities to mediate growth factors and cytokines, stem cell therapy could serve as a promising approach for difficult-to-heal diabetic wounds. Herein, we review recent literatures on stem cell-based therapies and propose that the cautiously administration of stem cells, such as the laboratory expandable pluripotent iPS cells or especially the easily obtainable autologous ASCs or its secretomes, would be a powerful implement to accelerate diabetic non-healing. In other words, the management of stem cells should be considered for diabetic wound therapeutics or at least as a part of a combination therapy.

## Figures and Tables

**Table 1:** The roles of major growth factors and cytokines in diabetic wound healing. (Numbers in the parentheses denote the reference numbers).

Growth Factors/Cytokines	Roles in Diabetic Wound Healing
TNF-α [36,37]	Immunomodulation Sustained inflammation Disturb cell migration and re-epithelization
TGF-β [38,39,40,41,42,43,44,45,46]	Immunomodulation Promote cell proliferation and migration Improve epithelialization, angiogenesis, and extracellular matrix synthesis Regulate other growth factors Recruit progenitor cells
EGF [47,48,49]	Promote cell proliferation and differentiation Regulate various downstream signaling pathways Anti-inflammatory effects
VEGF [50,51,52]	Promote angiogenesis Promote cell proliferation and migration Suppress apoptosis Improve vascularization, epithelialization, and collagen deposition
PDGF [53,54,55,56,57]	Promote cell proliferation Promote angiogenesis Regulate various downstream signaling pathways Regulate oxidative balance
FGF [58,59,60,61]	Promote cell proliferation, differentiation and survival Promote angiogenesis Regulate blood glucose metabolism
Interleukins [59,62,63,64,65]	Inflammatory molecules in the process of wound healing particularly in inflammation (most ILs) Improve vascularization, re-epithelialization, and granulation tissue formation (IL-22) Promote angiogenesis and collagen deposition (IL-25) Maintain glucose homeostasis (IL-25)

**Table 2:** Benefits and limitations of various types of stem cell in diabetic wound healing. (Numbers in the parentheses denote the reference numbers).

Stem Cell Type	Benefits	Limitations
Embryonic stem cells (ESCs) [79,80,81,82,83,84,85,86,87]	Pluripotency	Ethical, religious, and legal issues (cells are isolated from living embryo)
Induced pluripotent stem cells (iPSCs) [88,89,90,91,92,93]	Pluripotency No ethical concerns Less immunological rejection Laboratory expandable from source cells	Cancerous risks Low cell reprogramming efficiency High processing costs
Mesenchymal stem cells (MSCs) [94,95,96,97,98,99,100,101,102,103,104,105,106]	Multipotent No ethical concerns No immunological rejection Immunomodulation of paracrine signaling	Less self-renewal ability (compared to ESCs and iPSCs) Limited differentiated ability (only to tissue-forming cell lineages) Invasive isolation procedure (if from bone marrow)
Adipose-derived stem cells [107,108,109,110]	Multipotent No ethical concerns No immunological rejection Immunomodulation of paracrine signaling Availability (abundant and easily obtainable) Secretomes (cell free system available)	Short lifespan Less self-renewal ability (compared to ESCs and iPSCs) Limited differentiated ability (only to tissue-forming cell lineages) Donor specificities influence stem cell’s potency and quality

## Data Availability

Not applicable.
